# Endocannabinoid System Regulation in Female Rats with Recurrent Episodes of Binge Eating

**DOI:** 10.3390/ijms232315228

**Published:** 2022-12-03

**Authors:** Mariangela Pucci, Claudio D’Addario, Emanuela Micioni Di Bonaventura, Francesca Mercante, Eugenia Annunzi, Federico Fanti, Manuel Sergi, Luca Botticelli, Giacomo Einaudi, Carlo Cifani, Maria Vittoria Micioni Di Bonaventura

**Affiliations:** 1 Faculty of Bioscience and Technology for Food, Agriculture and Environment, University of Teramo, Via Renato Balzarini 10, 64100 Teramo, TE, Italy; 2 Pharmacology Unit, School of Pharmacy, University of Camerino, Via Madonna delle Carceri 9, 62032 Camerino, MC, Italy; 3 Department of Neuroscience, Imaging and Clinical Sciences, University “G. d’ Annunzio” of Chieti-Pescara, Via dei Vestini 33, 66100 Chieti, CH, Italy

**Keywords:** binge eating, endocannabinoid system, epigenetic mechanisms, food restriction, frustration stress, gene expression

## Abstract

Recurrent Binge Eating (BE) episodes characterize several eating disorders. Here, we attempted to reassemble a condition closer to BE disorder, and we analyzed whether recurrent episodes might evoke molecular alterations in the hypothalamus of rats. The hypothalamus is a brain region which is sensitive to stress and relevant in motivated behaviors, such as food intake. A well-characterized animal model of BE, in which a history of intermittent food restriction and stress induce binge-like palatable food consumption, was used to analyze the transcriptional regulation of the endocannabinoid system (ECS). We detected, in rats showing the BE behavior, an up-regulated gene expression of cannabinoid type-1 receptor (CB1), sn-1-specific diacylglycerol lipase, as well as fatty acid amide hydrolase (*Faah*) and monoacylglycerol lipase. A selective reduction in DNA methylation was also observed at the promoter of *Faah*, which is consistent with the changes in the gene expression. Moreover, BE behavior in rats was associated with an increase in anandamide (AEA) levels. Our findings support the relevant role of the ECS in the regulation of food intake in rats subjected to repeated BE episodes, and, in particular, on AEA signaling, acting via CB1 and FAAH modulation. Notably, the epigenetic regulation of the *Faah* gene might suggest this enzyme as a possible target for developing new therapeutical approaches.

## 1. Introduction

The phenomenon of compulsive overeating, or Binge Eating (BE), is characterized by eating a large amount of food in a short period of time, and is associated with a loss of control when it occurs. Recurrent BE episodes characterize several eating disorders, such as Binge Eating Disorder (BED), the most prevalent of all eating disorders [1]; Bulimia Nervosa (BN); and the binge/purging subtype of Anorexia Nervosa (AN) [2]. The main difference between BN and BED is the presence of compensatory behaviors after the BE episode, which occur in individuals with BN, while they are absent in the subjects with BED [2].

It should be highlighted that during the COVID-19 pandemic, the increased psychosocial stressors exacerbated pre-existing eating disorders and the vulnerability to develop them [3]. Negative emotions affect and trigger episodes of BE [4], and the pandemic situation itself contributed to a worsening of the eating disorders enhancing the BE behavior [5]. It is estimated that there was an increased prevalence of eating disorders, particularly in women: ranges of lifetime between 2000 and 2018 have been reported to be 3.3–18.6% for women and 0.8–6.5% for men [6]; specifically, BED affects 1.5% of women and 0.3% of men worldwide. BED was introduced for the first time as an Eating Disorder not Otherwise Specified in the 4th edition of the Diagnostic and Statistical Manual of Mental Disorders (DSM-IV; APA, 1994), finally becoming an autonomous eating disorder in the DSM-V [2]. Notably, BED is associated with additional health risks, such as diabetes, heart disease, obesity, and medical and psychiatric comorbidities [7,8].

Both BN and BED are complex disorders, meaning that several factors, (biological, social, and environmental) should be considered to understand their development [9,10]. These factors may interact to facilitate their progression, influencing gene functions, and in this frame, the study of epigenetic mechanisms is of clear relevance. It is known that gene expression is temporary, and how long it actually lasts depends on the specifics of the process and on the impact of environmental factors on the progression of various diseases, including BN and BED [11,12,13].

Different genetic loci are environmentally sensitive and, through epigenetic modifications, their transcriptional regulation might be involved in the risk of developing an eating disorder. So far, candidate genes have been proposed as epigenetically modified in eating disorders [14], and endogenous signaling pathways have been already found to be epigenetically modulated in eating disorders [12,13,15]. We have already reported that epigenetic mechanisms play a role in the effects evoked by BE behavior [11,12,13]. In this work, we focused on the study of the endocannabinoid system (ECS), which is well-known to play a role in eating behaviors [16]. The components of this system have been reported to be epigenetically regulated in different pathological conditions [17,18].

Anandamide (N-arachidonoylethanolamine, AEA) and 2-arachidonoylglycerol (2-AG) represent the most active class of endogenous cannabinoids (eCBs). They are produced by multiple synthetic pathways, starting from lipid precursors present in cell membranes, only when and where they are needed following stimuli. Selective metabolic enzymes, such as N-acyl-phosphatidylethanolamines hydrolyzing phospholipase D (NAPE-PLD) and sn-1-specific diacylglycerol lipase (DAGL), as well as fatty acid amide hydrolase (FAAH) and monoacylglycerol lipase (MAGL), are involved in the biosynthesis and degradation of AEA and 2-AG, respectively. Together with the enzymatic machinery, ECS is comprised of cannabinoid type-1 (CB1) and cannabinoid type-2 (CB2) receptors, both of which are representative of the inhibitory G-protein coupled receptor (GPCR) family (for recent reviews, see [19,20]).

We and others have already reported the transcriptional regulation of ECS in preclinical and clinical conditions of different eating disorders [20,21,22,23], as well as in food addiction [24] and obesity [25,26]. In particular, in a very well-characterized model of a BE episode [27,28], we reported, among the different ECS components, a selective down-regulation of *Faah* gene expression accompanied by an increase in histone 3 acetylation at lysine 9 in the hypothalamus of female rats in which a BE episode occurred [13]. In this animal model, the bingeing rats which had been previously exposed to repeated food restriction plus frustration stress (see Cifani et al. [27] and methodological paragraph below) showed a significant increase in palatable food consumption during the 2 h test compared to the other experimental groups. Taking into account these previous results in the hypothalamus, a brain region sensitive to stress and relevant in motivated behaviors such as food intake, this work aims to ascertain further alterations in the ECS system after repeated BE episodes in the same area already investigated [29,30]. To achieve this goal, we used the aforementioned BE paradigm [27], in which the rats developed the BE behavior every time they were subjected to caloric restrictions plus stress procedures and palatable food availability. Thus, this rat model offers us the opportunity to mimic regular and recurrent BE episodes, resembling individuals with BED, and to analyze the possible mechanisms involved in the transcriptional regulation of the ECS.

## 2. Results

### 2.1. BE Behavior in Rats Exposed to Food Restrictions Plus Stress

**Figure 1:**
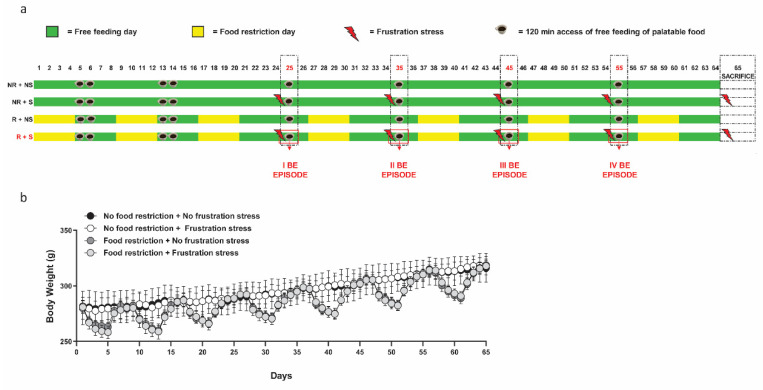
Schematic representation of BE model (**a**). Mean ± SEM (**b**) of body weight (grams) for the entire duration of the study; n = 6 per group. NR + NS: Non-restricted and Non-stressed rats; NR + S: Non-restricted and Stressed rats; R + NS: Restricted and Non-stressed rats; R + S: Restricted and Stressed rats.

Figure 1a schematically represents the BE model (described in detail in the Supplementary Materials) and shows the four experimental groups that we defined as follows: NR + NS: Non-restricted and Non-stressed rats; NR + S: Non-restricted and Stressed rats; R + NS: Restricted and Non-stressed rats; R + S: Restricted and Stressed rats. Only in the last group, R + S rats, did the BE behavior arise, triggered by repeated restrictions of standard food in addition to stress, during which the rats can see and smell the familiar palatable food without having access to it for 15 min [27,31]. Thus, in our model, an episode of “binge eating” is operationally defined as a significantly higher palatable food consumption during the 2 h test in the R + S rats than in the other groups. This maladaptive eating behavior is measured by the palatable food consumed in 2 h, which is significantly higher in R + S than in the other experimental groups. It is not limited to a single episode, but is also maintained over time. In fact, in this work, the R + S group continued to show BE behavior, specifically four episodes of BE. In the fifth episode, they were sacrificed immediately after stress and before eating palatable food, and brains were collected. Between each BE episode, after 1 day off (free feeding day), rats were exposed to 4 days of standard food restriction, followed by 4 days of free chow availability (see Figure 1a and Figure S1), in order to recover the previously lost body weight [32,33,34], before being given access to palatable food. Figure 1b highlights these body weight fluctuations during the alternations of restriction cycles, as well as the subsequent lack of statistical difference (*p* > 0.05) in the body weight of the rats, in the restricted or non-restricted groups, on each feeding test day (days: 25, 35, 45, 55, and 65).

The significantly increased palatable food intake (*p* < 0.01) in R + S rats compared to the other groups, during the first 15 min and the total 120 min of each feeding test, is summarized in Table 1. Specifically, two-way ANOVA showed a significant interaction between the two factors (restriction and stress) for each test at both time points (the first test: 15 min [F(1, 17) = 17.83, *p* < 0.01] and 120 min [F(1, 17) = 15.63, *p* < 0.01]; the second test: 15 min [F(1, 16) = 65.71, *p* < 0.01] and 120 min [F(1, 16) = 21.91, *p* < 0.01]; the third test: 15 min [F(1, 17) = 8.56, *p* < 0.01] and 120 min [F(1, 17) = 12.19, *p* < 0.01]; the fourth test: 15 min [F(1, 17) = 16.69, *p* < 0.01] and 120 min [F(1, 17) = 17.69, *p* < 0.01].

### 2.2. Regulation of ECS in Rats Exposed to Food Restrictions and Stress Procedure

**Figure 2:**
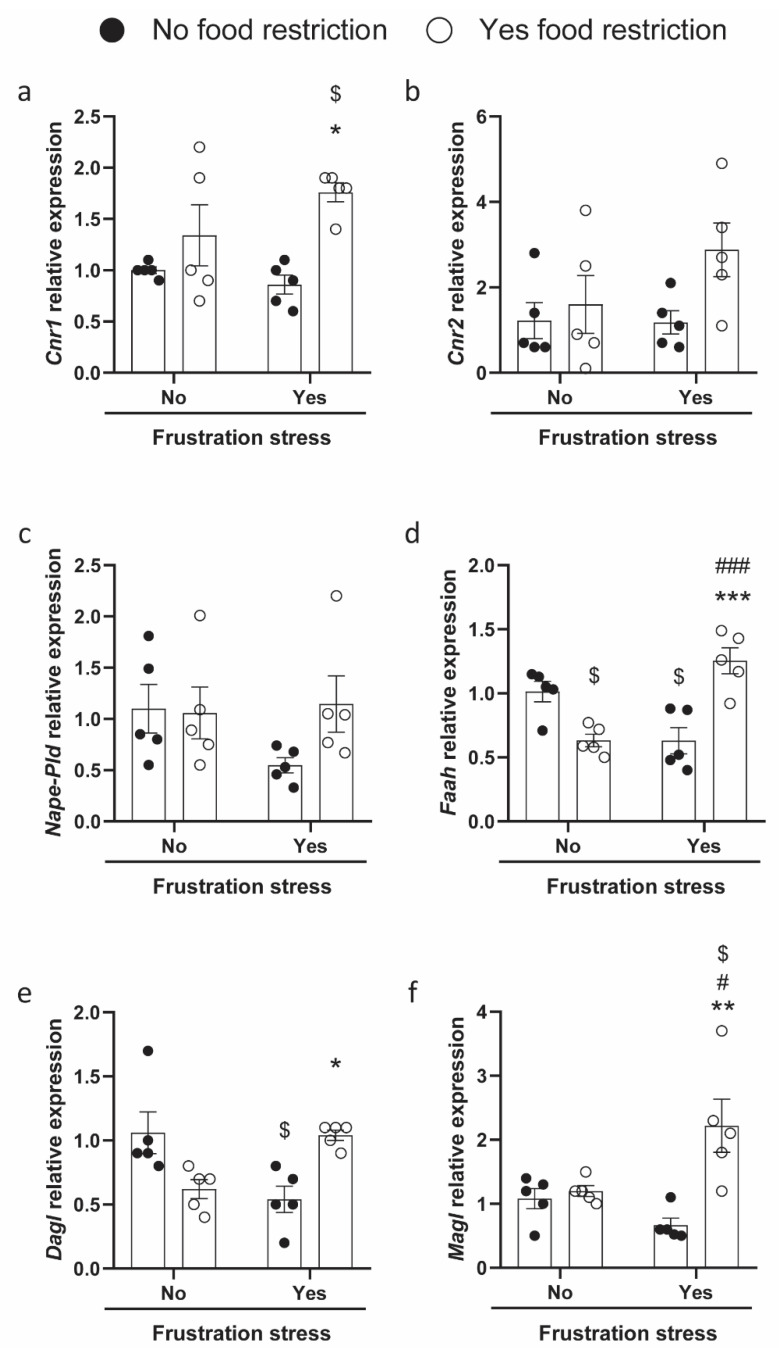
ECS relative gene expression in the hypothalamus of rats exposed (or not) to restriction and stress (NR + NS: Non-restricted and Non-stressed rats; NR + S: Non-restricted and Stressed rats; R + NS: Restricted and Non-stressed rats; R + S: Restricted and Stressed rats) reported as 2^−ΔΔCt^ values calculated by Delta–Delta Ct (ΔΔCt) method versus NR + NS animals posed equal to 1. Expression was normalized using an average CT-value from both reference genes (GAPDH and β-ACT), and data are reported as mean ± SEM; n = 5 per group. Relative expression of: (**a**) *Cnr1* (cannabinoid type-1); (**b**) *Cnr2* (cannabinoid type-2); (**c**) *Nape-Pld* (N-acyl-phosphatidylethanolamines hydrolyzing phospholipase D; (**d**) *Faah*, (fatty acid amide hydrolase); (**e**) *Dagl*, (sn-1-specific diacylglycerol lipase); (**f**) *Magl*, (monoacylglycerol lipase). Data were analyzed by two-way ANOVA in a 2 (food restriction: no, yes) × 2 (frustration stress during testing: no, yes) factorial design. Bonferroni’s post hoc tests were used to follow up on significant interaction or main effects (*p* < 0.05) from the factorial ANOVAs. Significant differences are indicated: ^$^ *p* < 0.05 versus NR + NS, ^#^ *p* < 0.05 versus R + NS, ^###^ *p* < 0.001 versus R + NS, * *p* < 0.05 versus NR + S, ** *p* < 0.01 versus NR + S, *** *p* < 0.001 versus NR + S.

In order to evaluate whether recurrent episodes of BE evoke changes in the regulation of ECS gene expression, we analyzed the mRNA levels as well as the potential epigenetic modulation in the hypothalamic region of all the experimental groups. Standard food restriction cycles in combination with stress determined changes in the gene expression of *Cnr1* (the gene coding for the cannabinoid receptor type 1, CB1), *Faah*, *Dagl,* and *Magl*. All the observed changes were reported in Figure 2. Two-way ANOVA showed that *Cnr1* mRNA levels were affected by food restriction [F(1, 16) = 14.40, *p* = 0.0016], but not by stress [F(1, 16) = 0.734, *p* = 0.404], without a significant interaction between these two factors [F(1, 16) = 2.936, *p* = 0.106]. Bonferroni’s post hoc test showed a significant increase in the *Cnr1* mRNA level in R + S rats, and post hoc group differences are reported in Figure 2a. Moreover, significative changes in the gene expression of *Faah* were observed in the same experimental group, and two-way ANOVA showed that the increase was not affected by food restriction [F(1, 16) = 1.994, *p* = 0.177] or stress [F(1, 16) = 1.928, *p* = 0.184], but by a significant interaction between these two factors [F(1, 16) = 34.45, *p* < 0.001]. Post hoc group differences are indicated in Figure 2d. Significant changes were also observed in the gene expression of metabolic enzymes involved in the biosynthesis and degradation of 2-AG (Figure 2e,f). Two-way ANOVA showed that *Dagl* mRNA levels were not affected by food restriction [F(1, 16) = 0.0814, *p* = 0.780] or stress [F(1, 16) = 0.226, *p* = 0.641], but by a significant interaction between these two factors [F(1, 16) = 19.99, *p* < 0.001]. *Magl* gene expression was affected by food restriction [F(1, 16) = 11.62, *p* = 0.004] and not by stress [F(1, 16) = 2.118, *p* = 0.167], but rather by a significant interaction between these two factors [F(1, 16) = 8.645, *p* < 0.011]. Post hoc differences are indicated in Figure 2e,f.

**Figure 3:**
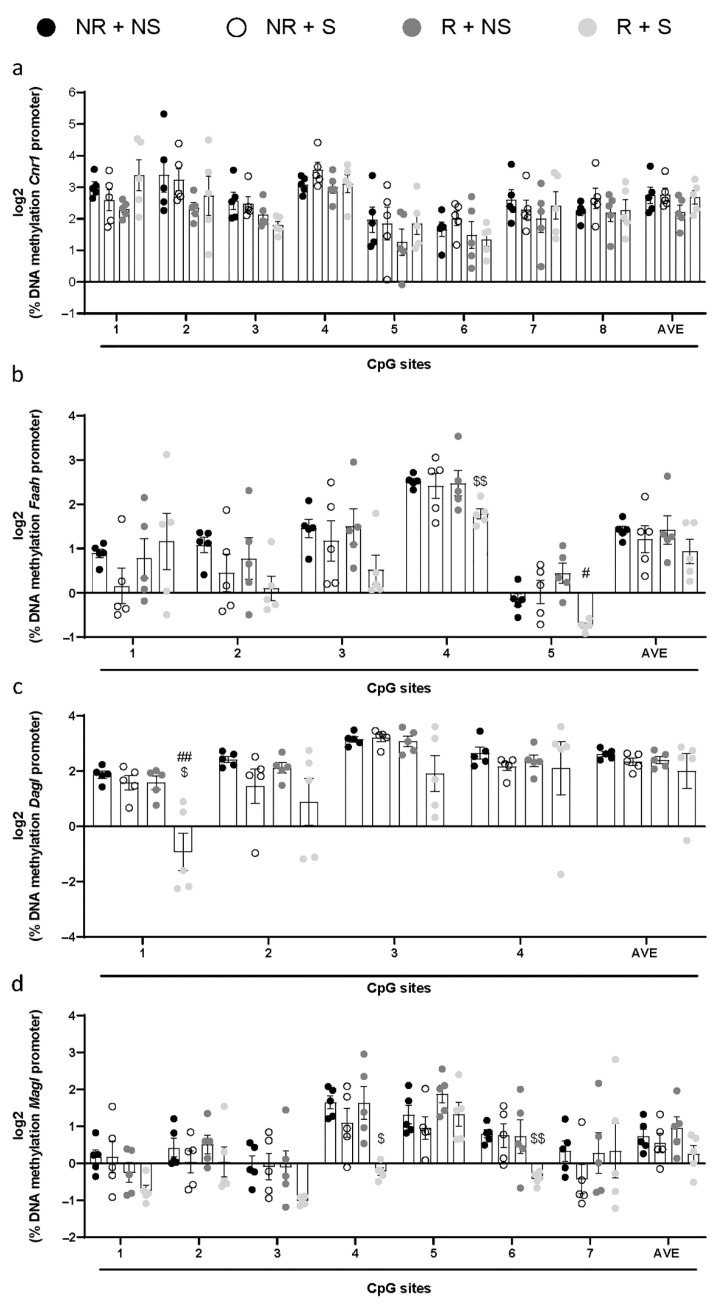
Amount of methylated DNA at *Cnr1* (**a**), *Faah* (**b**), *Dagl* (**c**), and *Magl* (**d**) promoter regions in the hypothalamus. Values on the *y*-axis represent, in log2 scale, the % of methylation values of individual CpG sites under study, as well as of the average (AVE) of all CpG sites ± SEM; n = 5 per group. Data were analyzed by multiple *t*-test and corrected for multiple comparisons using the Holm–Sidak method. Significant differences are indicated: ^$^ *p* < 0.05 versus NR + NS, ^$$^ *p* < 0.01 versus NR + NS, ^#^ *p* < 0.05 versus R + NS, ^##^ *p* < 0.01 versus R + NS.

**Figure 4:**
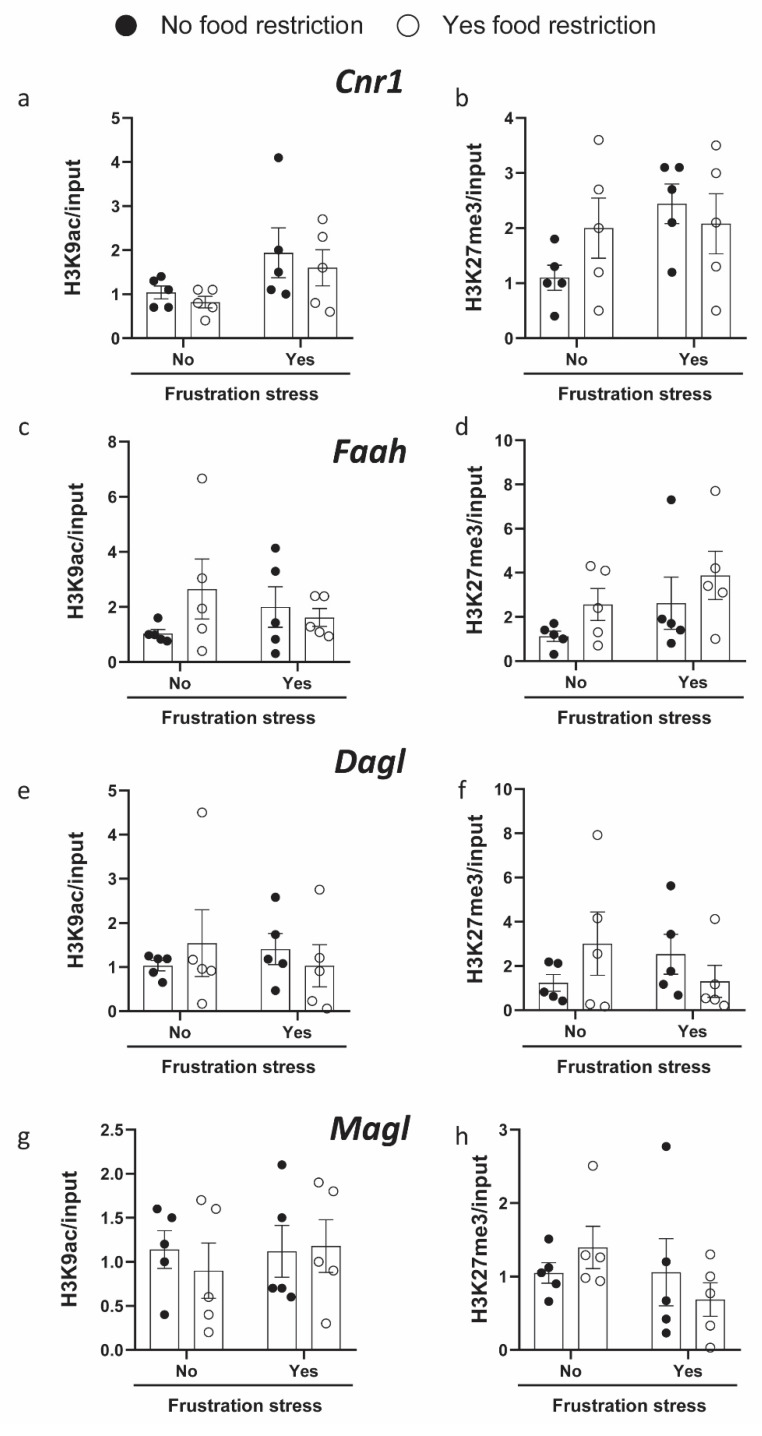
*Cnr1* (**a**,**b**), *Faah* (**c**,**d**), *Dagl* (**e**,**f**) and *Magl* (**g**,**h**) promoter histone modifications. RT-qPCR analyses of H3K9ac (**a**,**c**,**e**,**g**) and H3K27me3 (**b**,**d**,**f**,**h**) immunoprecipitated DNA fragments at promoter in rat hypothalamus. ChIP histogram shows the levels of specific histone modifications, normalized to total input DNA, in rats exposed (or not) to restriction and stress. Data were expressed as means ± SEM, n = 5 per group. Data were analyzed by two-way ANOVA in a 2 (food restriction: no, yes) × 2 (frustration stress during testing: no, yes) factorial design. Bonferroni’s post hoc tests were used to follow up on significant interactions or main effects from the factorial ANOVAs.

In order to evaluate the potential involvement of epigenetic mechanisms in the regulation of gene expression in the BE model, we analyzed DNA methylation (Figure 3) and histone modification (Figure 4) at the promoter regions in rat hypothalamus.

DNA methylation did not show a significant difference in all combined CpG sites, nor in methylation of each single site, examined in the promoter region of *Cnr1*. However, a significative decrease in DNA methylation at the *Faah* promoter was detected in the R + S group on the fourth and fifth CpG sites which were valued. Multiple *t*-test, corrected for multiple comparison using the Holm–Sidak method, showed a decrease in the percentage of DNA methylation in the CpG sites 4 (*p* = 0.007 versus NR + NS) and 5 (*p* = 0.031 versus R + NS). A significant change in DNA methylation was also observed in the first CpG site on the *Dagl* promoter, and multiple *t*-test, corrected by the Holm-Sidak multiple comparison, indicated a significant decrease at CpG site 1 of the R + S group (CpG site 1, *p* = 0.005 versus NR + NS and *p* = 0.017 versus R + NS). Moreover, we observed a significant decrease in the fourth (CpG site 4, *p* = 0.024 versus NR + NS) and sixth (CpG site 6, *p* = 0.004 versus NR + NS) CpG sites analyzed in the promoter region of *Magl*.

Moreover, we observed a significant inverse correlation between *Faah* gene expression (2(−ΔΔCt) values) and DNA methylation, considering the fifth CpG sites analyzed (Spearman’s r = − 0.5634, *p* = 0.0120) (Table 2 and Figure S3).

The analysis of H3 modification at the *Cnr1* gene promoter showed that H3K9Ac levels were affected by stress [F(1, 16) = 5.339, *p* = 0.0345], but not by restriction [F(1, 16) = 0.5933, *p* = 0.4524], without a significant interaction between these two factors [F(1, 16) = 0.027, *p* = 0.8710]. H3K27me3 levels were not significantly different in the *Cnr1* promoter region between all groups in the examined area (Figure 4).

Bonferroni’s multiple comparison test did not report a significant change in H3K9Ac at the promoter region in stressed rats which had undergone restriction, compared with all the other experimental groups. H3K27me3 levels were not significantly affected by stress nor by restriction in the *Cnr1* promoter region. The analysis of H3 modifications did not show significant changes in H2K9Ac and H3K27me3 in *Faah*, *Magl,* or *Dagl* promoter regions among the experimental groups.

### 2.3. Endogenous Content of eCBs

**Figure 5:**
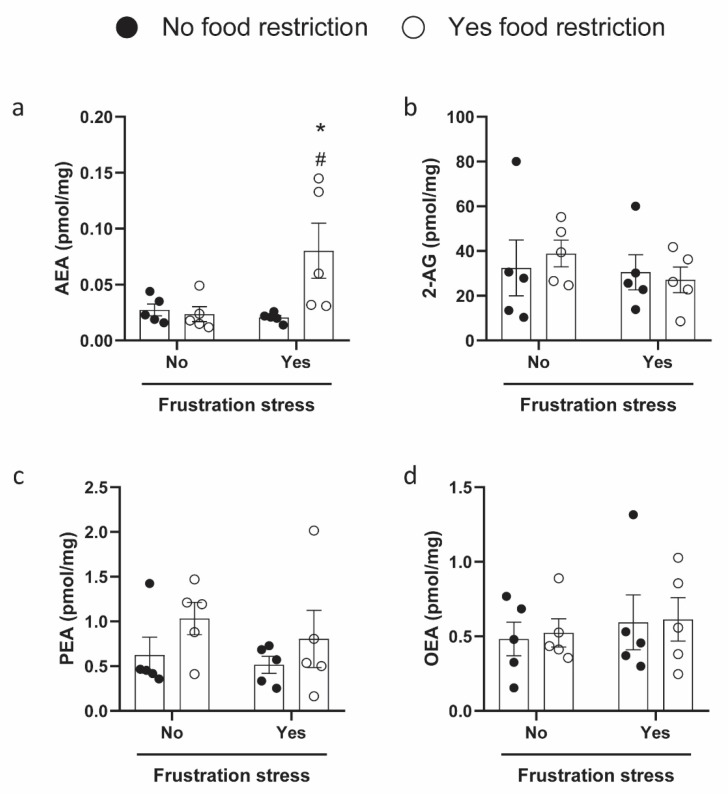
Endogenous levels of eCBs ((**a**) AEA, Anandamide; (**b**) 2-AG, 2-arachidonoylglycerol; (**c**) PEA, N-palmitoylethanolamine and (**d**) OEA, N-oleoylethanolamide) in the hypothalamic region of rats exposed or not to BE protocol. Data were expressed as means ± SEM, n = 5 per group. Data were analyzed by two-way ANOVA in a 2 (food restriction: no, yes) × 2 (frustration stress during testing: no, yes) factorial design. Bonferroni’s post hoc tests were used to follow up on significant interaction or main effects (*p* < 0.05) from the factorial ANOVAs. Significant differences are indicated: ^#^ *p* < 0.05 versus R + NS, * *p* < 0.05 versus NR + S.

Finally, the endogenous levels of the major eCBs, AEA and 2-AG, and the major eCB-like compounds, N-palmitoylethanolamine (PEA) and N-oleoylethanolamide (OEA), were measured in the hypothalamic region of rats exposed or not to restriction and stress by means of liquid chromatography–mass spectrometry (UHPLC-MS/MS) (Figure 5). It was found that the AEA content markedly changes in R + S animals. Two-way ANOVA showed that AEA levels were affected by food restriction [F(1, 16) = 4.561, *p* = 0.048], but not by stress [F(1, 16) = 3.633, *p* = 0.075], and that a significant interaction between these two factors was present [F(1, 16) = 5.888, *p* = 0.027]. Post hoc comparisons using Bonferroni’s multiple tests revealed a significant increase in AEA levels in the R + S group, with respect to R + NS (*p* = 0.0445) and NR + S (*p* = 0.0317).

Surprisingly, a direct correlation between AEA levels and *Faah* gene expression was observed (Spearman’s r = 0.4842, *p* = 0.0305), while an inverse correlation was observed between AEA levels and *Faah* DNA methylation at CpG site 5 (Spearman’s r = −0.5353, *p* = 0.0182) (Table 3 and Figure S3). Moreover, a direct correlation between the levels of AEA and the palatable food intake, calculated as a mean of 120-min time point palatable food intake of the four feeding tests in each experimental group, was also observed (Spearman’s r = 0.6045, *p* = 0.0048) (Table 3 and Figure S3).

## 3. Discussion

The first relevant result of this work is the induction of repeated BE episodes in rats. In fact, once established, the BE behavior occurred once every 9 days, triggered by the combination of standard food restriction and food-related stress, as was previously carried out in other animal models where the BE episode was repeated over time [35,36]. However, the choice of model of Cifani et al. [27] was driven by the well-known role of the dieting and stress combination in the development of eating disorders [37,38], particularly of BED [2,39,40,41,42,43,44,45,46], and that caloric restriction anticipates the stress-induced susceptibility to BE in the normal population [46,47,48,49,50,51,52,53,54].

From a molecular point of view, we report an increase in CB1 gene expression in the hypothalamus of rats showing repeated BE behaviors. It is already well-known that hypothalamic CB1 receptor signaling is a key determinant of energy expenditure [55], with a crucial role in the reinforcement and motivational properties of highly palatable food [56,57,58]. In fact, CB1 receptors are abundant in the hypothalamic nuclei, controlling food intake [59,60,61,62], and infusion of CB1 receptor agonists into different hypothalamic nuclei increases food intake [61,63,64,65].

Mice lacking the CB1 receptor gene (CB1-KO) display reduced rewarding behaviors [66], and among the different brain regions, the hypothalamus appears to be relevant in these effects [67,68]. This suggests that an increased food intake could be driven by an external stimulus (i.e., the stressful procedure related to food in addition to the restriction in our model) induced by the activation of the lateral hypothalamus [69].

In this study, the altered levels of *Cnr1* mRNA observed in the BE group were accompanied by selective higher hypothalamic levels of AEA, whereas no changes were observed for the other eCBs analyzed. It has already been reported that eCB levels are strongly modulated by the feeding status of the animals [61,70,71,72], and the enhanced levels of AEA might facilitate the compulsivity to eat, thus evoking the BE behavior, as already suggested in women with BED [73]. No changes in 2-AG have been found in BED patients [73], nor in obese subjects [74], where instead AEA resulted increased just before the meal, allowing the authors to suggest a role for AEA as a physiological meal initiator [74].

In patients with BED, it has been also observed that AEA plasma levels decreased after eating the non-favorite food and significantly increased after eating the favorite food; while 2-AG plasma levels did not change in these conditions [22]. Moreover, the authors demonstrated that AEA levels were positively correlated to the subjects’ sensations of the urge to eat, allowing them to hypothesize the role of eCBs for the “wanting” and “liking” in food reward, and thus for their BE behavior [22].

Notably, the expression of enzymes responsible for the eCBs synthesis (DAGL and NAPE-PLD even if the latter not significantly) and degradation (MAGL and FAAH) was found to be up-regulated. It might be hypothesized that if there is a need of more eCBs, contextually, this boost might lead to compensatory mechanisms. Our data might appear in contradiction with previous studies showing that the inhibition of the enzyme enhances the motivation for food intake [13,75,76]. However, these studies were conducted in animals deficient in FAAH [76], with inhibition of the enzyme [75], or, with regard to our previous data, following a single BE episode [13]. We now report an increase in *Faah* gene expression, showing that, after repeated BE episodes, the production of AEA is still high, but the system is reacting to counteract these altered AEA levels and producing more enzyme than necessary for its degradation. It would be necessary in future studies to analyze whether these changes occur differently following additional BE episodes.

When we analyzed the possible role of epigenetic mechanisms in the regulation of ECS gene expression levels, it was observed that only DNA methylation appears to be involved, and only for the two degrading enzymes, where specific CpG sites were hypomethylated in the BE group. Again, this is different from what we observed following a single BE episode, where the only epigenetic modification was the reduction in the BE rats at the level of the *Faah* gene for histone 3 acetylation at lysine 9. We now show that repeated BE episodes induce a reduction in DNA methylation at selective CpG sites at *Faah*, as well as *Dagl* and *Magl* gene promoters. Previous human studies have already reported alterations in DNA methylation at the *FAAH* promoter in Alzheimer’s Disease [77] and alcoholism [78]. Instead, the epigenetic modulation of *Magl* and *Dagl,* so far, has not been reported. These alterations, which are also associated with the increases in gene expression, are consistent with the unaltered 2-AG levels in our experimental conditions, meaning that as long as the eCB is produced, it seems to be immediately degraded. Based on previous results, in which a selective alteration of the *Faah* gene was observed in the Hypothalamus of female rats that showed BE episodes [13], in this work, we confirmed the molecular alteration of ECS in the same brain region after recurrent episodes of binge eating. However, further studies would be necessary to extend the investigation into other brain regions, in order to better characterize the role of ECS in the rewarding aspect of food intake and its involvement in BE behavior.

In conclusion, our data support the relevant role of the ECS in the regulation of food intake in the hypothalamus of rats subjected to repeated BE episodes, as well as in AEA signaling via CB1 and FAAH modulation. Moreover, the epigenetic regulation of the *Faah* gene might suggest this enzyme as a possible target for developing new therapies taking advantage of the reversibility of epigenetic alterations.

## 4. Materials and Methods

### 4.1. Subjects and Diet Composition

Twenty-four female Sprague–Dawley rats (Charles River, Calco, Italy), weighing 225–250 g at the beginning of the experiments, were housed under a 12-h light/dark cycle (lights on at 9:00 a.m.), at constant temperature (20–22 °C) and humidity (45–55%), and with access to standard food and water ad libitum for 1 week before the experiments. Thereafter, they were divided into 4 groups (n = 6 per group). A detailed description of the BE model is reported in Supplementary Materials. In accordance with the higher prevalence of eating disorders among young adolescents and adult women rather than men, female rats were used [6,79], as in previously published works [80,81,82].

### 4.2. Tissue Collection, Nucleic Acids and Chromatin Extraction

Immediately after sacrifice, brains were rapidly removed, and the hypothalamus, obtained by dissection, was immediately frozen and stored at −80 °C until processing [15]. DNA and RNA were isolated from half of the hypothalamus according to the modified method by Chomczynski and Sacchi [83]. The quantity and purity were assessed using NanoDrop Spectrophotometer (Thermo Scientific, Waltham, MA, USA), while the RNA integrity was validated by running 1 µg in 1% agarose gel. The quality of RNA was indicated by the lack of a smear on the lower part of the gel, and by the presence of 28S ribosomal RNA twice as intense as that of 18S rRNA.

Chromatin was prepared from frozen tissues as previously described, with minor modifications [84]. Briefly, proteins were cross-linked to DNA by addition of formaldehyde at a final concentration of 1%, in phosphate-buffered saline containing protease inhibitor cocktail and sodium butyrate. Then, the cross-linking reaction was quenched by adding glycine. The sample was washed and lysed by resuspending with a pipette in 120 μL of lysis buffer [13,85]. The chromatin was sonicated to shear the DNA to fragments ranging in size from 150 to 700 bp, as analyzed by agarose gel electrophoresis [86]. At this point, the chromatin was stored at −80 °C until processing.

We used total RNA to assess the relative abundance of ECS genes by real-time qPCR, and DNA to analyze the % of methylation on the promoter region of the ECS gene by pyrosequencing. Histone modifications were assessed by chromatin immunoprecipitation. A detailed description of gene expression and DNA methylation analysis as well as the Chromatin immunoprecipitation is reported below, according to our previous published studies [11,12,13,87].

### 4.3. Analysis of Gene Expression

High-quality total RNA was converted to cDNA with a SensiFAST cDNA synthesis Kit (Meridian Bioscience, Cincinnati, OH, USA), using a unique blend of random hexamer and anchored oligo dT primers, according to the manufacturer’s guidelines. The cDNAs were subsequently diluted three times, and relative abundance of each mRNA species was assessed by qRT-PCR, employing 1 μL of the diluted samples in a final volume of 15 μL using Opticon 2 MJ Research (Biorad, Hercules, CA, USA). The primers used for the amplification are reported in Table S1. All of the data were normalized using an average CT-value from both endogenous reference gene β-actin and GAPDH. The use of two or more reference genes for normalization with the straightforward ΔΔCT calculation improves the reproducibility and robustness of comparative RT-qPCR-based gene expression analyses [88]. The rate of increased expression was calculated using the Livak (2^−ΔΔCT^) method [89].

### 4.4. DNA Methylation

The CpG islands were predicted using MethPrimer software. Briefly, the prediction is based on an algorithm which slides across the sequence at a specific shift value, examining the CG content and the ratio observed/expected (Obs/Exp) in a defined windows size. A CpG island is defined as a DNA stretch that is at least 100 bp-long, with a CG content >50% and an Obs/Exp ratio of CpG dinucleotides >0.6. DNA methylation analysis was determined on bisulfite converted DNA from hypothalamic rat brain tissues using pyrosequencing [11,12,26,87], a gold standard method for the identification of specific methylation patterns [90,91]. After extraction, 0.5 μg of DNA from each sample was treated with bisulfite, using the EZ DNA Methylation-Gold™ Kit (Zymo Research, Irvine, CA, USA). Bisulfite-treated DNA was amplified by PyroMark PCR Kit (Qiagen, Hilden, Germany) in accordance with the manufacturer’s protocol. PCR conditions were as follows: 95 °C for 15 min, followed by 45 cycles of 94 °C for 30 s, 56 °C for 30 s, 72 °C for 30 s, and, finally, 72 °C for 10 min. PCR products were verified by agarose electrophoresis. Pyrosequencing methylation analysis was conducted using the PyroMark Q24 (Qiagen, Hilden, Germany). The degree of methylation was analyzed using PyroMark Q24 Software (Qiagen, Hilden, Germany), which calculates the methylation percentage [mC/(mC + C)] for each CpG site, allowing for quantitative comparisons (mC is methylated cytosine, C is unmethylated cytosine). The sequences for the utilized primers are reported in Table S2.

### 4.5. Chromatin Immunoprecipitation

Chromatin–protein complexes were immunoprecipitated with 1 μg of anti-H3K9ac and anti-H3K27me3 antibodies and 20 μL of fully re-suspended protein A/G magnetic beads (Dynabeads® Protein A, Invitrogen, Carlsbad, CA, USA). Beads were then washed two times with sonication buffer, and DNA was eluted in elution buffer. Cross-links were reversed overnight. Immunoprecipitated and input DNA were purified by a DNA clean and concentrator kit (Zymo Research, Irvine, CA, USA), and the relative abundance was assessed by RT-qPCR, using the SensiFAST SYBR Low-ROX kit (Meridian Bioscience, Cincinnati, OH, USA) with the following program: 10 min at 95 °C for initial denaturation, 15 s at 95 °C, and 1 min at 60 °C for 40 cycles, followed by 5 min at 72 °C for final extension. Each sample was assayed in triplicate, and the fold enrichment ratio was calculated as the value of the ChIP sample versus the corresponding input sample. The primers used for these studies are listed in Table S3. After removing a few microliters to serve as ‘input’ DNA, for each immunoprecipitation, 8 μg of chromatin was diluted 10-fold in radioimmunoprecipitation assay (RIPA) buffer (10 mM Tris–HCl, pH 7.5, 1 mM EDTA, 0.5 mM ethylene glycol tetraacetic acid, 1 percent Triton X-100, 0.1 percent SDS, 0.1 percent nadeoxycholate, and 140 mM NaCl) containing protease cocktail inhibitorand incubated overnight by rotation for 2 h at 4 °C. This was performed with agitation either with no antibody, as control, or with 1–4 μg of antibody previously coated with Protein A beads (Invitrogen, Carlsbad, CA, USA), against either H3K27me3 (PA5-114540 ThermoFisher, MA, USA) and H3K9Ac (PA5-117092 ThermoFisher, MA, USA), or normal rabbit IgG (17-658; Millipore, Bedford, MA, USA) as a negative control. The beads and associated immune complexes were washed three times with RIPA buffer and once with Tris–EDTA buffer. The immune complexes were eluted with elution buffer (20 mM Tris–HCl, 5 mM EDTA, 50 mM NaCl, 1 percent SDS) containing proteinase K (50 μg/mL, Qiagen, Hilden, Germany) at 68 °C for 2 h, DNA was recovered by phenol extraction, and ethanol was precipitated and resuspended in 50 μL of sterile water. This procedure has been described in more detail elsewhere [84]. Thereafter, RT-qPCR quantification of the genomic sequences from the rat proximal promoter regions associated with the immunoprecipitated proteins was carried out. The primers used for PCR amplification were designed using PRIMER 3 software [92]. The relative expression of different transcripts was calculated by the ΔΔCt method and converted to a relative expression ratio (2^−ΔΔCt^) for statistical analysis [89]. All chromatin immunoprecipitation (ChIP) data were normalized to the input DNA amounts (Ct values of immunoprecipitated samples were normalized to Ct values obtained from input). In addition, results regarding DNA from the different groups were normalized to results obtained on DNA from the non-restricted and non-stressed group. Each ChIP experiment was repeated at least three times.

### 4.6. UHPLC-MS/MS Analysis of eCBs Levels

After sonication of brain tissue, suspended in methanol and containing deuterated internal standard (AEA-d8, PEA-d4, 2-AG-d8 and OEA-d4), the lipid fraction was extracted from the sample with chloroform/water (2:1 *v*/*v*). The organic phase was dried under gentle nitrogen stream and then subjected to a micro-solid phase extraction (µSPE) procedure [93] for rapid clean-up, by using OMIX C18 tips from Agilent Technologies (Santa Clara, CA, USA). UHPLC-MS/MS analysis was performed by a Nexera XR LC 20 AD UHPLC system (Shimadzu Scientific Instruments, Columbia, MD, USA), coupled with a Qtrap 4500 from Sciex (Toronto, ON, Canada) equipped with a Turbo V electrospray ionization source and operating in positive mode (ESI+). Separation of the analytes was performed by a Kinetex XB-C18 1.7 µm 100 × 2.1 mm column from Phenomenex (Torrance, CA, USA). Mobile phases were water and acetonitrile, both with a concentration of 0.01% *v*/*v* formic acid. The levels of AEA, 2-AG, and PEA were then calculated as pmoles per mg of tissue.

### 4.7. Statistical Analysis

Results were presented as mean ± SEM. Sample sizes were calculated by performing a power analysis with G*Power software 3.0.10. Specifically, the number of animals to be used has been calculated with an alpha level of 0.05 and a statistical power of 0.8. Analysis of the results was performed using GraphPad Prism 6 for Windows (GraphPad Software, San Diego, CA, USA). In vivo data were analyzed by two-way ANOVA, for repeated measures, in a 2 (food restriction: no, yes) × 2 (frustration stress during testing: no, yes) factorial design. Bonferroni’s post hoc tests were used to follow up on significant interactions of the main effects (*p* < 0.05) from the factorial ANOVAs for each feeding test. Since we previously observed that BE does not occur during the estrous phase [94,95], all female rats in the estrous phase in each experimental group during the four feeding tests (on days 25, 35, 45, and 55) were excluded from the results (for details, see Supplementary Materials).

Statistical differences in gene expression were determined by two-way ANOVA, followed by multiple comparison or Bonferroni’s post hoc tests. DNA methylation at each CpG site was analyzed using multiple *t*-test, and corrected for multiple comparison using the Holm–Sidak method. In Table 2 and Table 3, data are compared by Spearman’s rank correlation coefficient. In all instances, the threshold for statistical significance was set at *p* < 0.05. In order to estimate the relationship between the variables, a simple linear regression analysis was performed (Table 2 and Table 3, and Figure S2).

## Figures and Tables

**Table 1:** Mean ± SEM of palatable food intake (kcal/kg) at 15- and 120-min time points during the four feeding tests in each experimental group: NR + NS: Non-restricted and Non-stressed rats; NR + S: Non-restricted and Stressed rats; R + NS: Restricted and Non-stressed rats; R + S: Restricted and Stressed rats. ** *p* < 0.01, different from the other three groups in each feeding test; n = 5–6 per group. Data were analyzed by two-way ANOVA in a 2 (food restriction: no, yes) × 2 (frustration stress during testing: no, yes) factorial design. Bonferroni’s post hoc tests were used to follow up on significant interaction or main effects (*p* < 0.05) from the factorial ANOVAs for each feeding test.

Feeding Tests	NR + NS		NR + S		R + NS		R + S	
	15 min	120 min	15 min	120 min	15 min	120 min	15 min	120 min
**FIRST**	84.1 ± 5.6	113.2 ± 9.5	70.6 ± 6.8	103.0 ± 12.7	87.1 ± 7.4	118.5 ± 8.0	131.3 ± 7.1 **	177.8 ± 4.4 **
**SECOND**	82.3 ± 7.4	120.7 ± 10.2	72.5 ± 3.0	101.3 ± 5.9	74.6 ± 5.2	117.4 ± 6.5	127.5 ± 4.5 **	166.1 ± 10.0 **
**THIRD**	73.1 ± 5.8	105.7 ± 7.8	78.9 ± 5.6	107.9 ± 5.5	87.0 ± 4.8	116.3 ± 5.0	132.2 ± 10.0 **	179.4 ± 14.4 **
**FOURTH**	72.3 ± 3.9	107.3 ± 1.0	83.6 ± 4.6	113.7 ± 3.1	86.6 ± 3.9	109.7 ± 3.6	130.7 ± 3.6 **	167.0 ± 9.6 **

**Table 2:** Correlation analysis between the percentage of DNA methylation and gene expression in *Faah*, *Dagl,* and *Magl* observed in rat hypothalamus. Data were compared by Spearman’s rank correlation coefficient, and r and *p* values are reported.

% DNA Methylation	Relative Gene Expression			
	Spearman’s r	*p* Value	Equation	*p* Value
*Faah* CpG site 4	−0.4404	0.059	Y = −2.853 × X + 7.706	0.0662
*Faah* CpG site 5	−0.5634	0.0120	Y = −0.7290 × X + 1.643	0.0179
*Dagl* CpG site 1	−0.258	0.301	Y = −0.7624 × X + 3.216	0.4919
*Magl* CpG site 4	−0.1685	0.5300	Y = −0.6307 × X + 3.654	0.3297
*Magl* CpG site 6	−0.1694	0.5121	Y = −0.2996 × X + 1.994	0.2883

**Table 3:** Correlation analysis between the levels of AEA and the percentage of DNA methylation and gene expression in *Faah* and *Cnr1*. Correlation analysis between gene expression of *Faah* and *Cnr1*. Correlation analysis between the levels of AEA and the palatable food intake (kcal/kg, calculated as a mean of 120-min time point palatable food intake of the four feeding tests) in each experimental group. Data were compared by Spearman’s rank correlation coefficient; *p* and r values are reported.

	**AEA levels**			
**% DNA Methylation**	**Spearman’s r**	***p* value**	**Equation**	***p* value**
*Faah* CpG site 4	−0.3561	0.1345	Y = −23.47 × X + 6.111	0.0826
*Faah* CpG site 5	−0.5353	**0.0182**	Y = −4.495 × X + 1.179	0.1065
**Relative gene expression**				
*Faah*	0.4842	**0.0305**	Y = 0.05542 × X − 0.01095	0.0288
*Cnr1*	0.4055	0.0761	Y = 0.02809 × X + 0.003122	0.1044
**Relative gene expression**	**Relative gene expression *Faah***			
	**Spearman’s r**	***p* value**	**Equation**	***p* value**
*Cnr1*	0.3796	0.0987	Y = 0.2759 × X + 0.5403	0.0678
**AEA levels**	**Palatable food intake (kcal/kg)**			
	**Spearman’s r**	***p* value**	**Equation**	***p* value**
	0.6045	**0.0048**	Y = 535.0 × X + 107.8	**0.0013**

## Data Availability

Not applicable.

## Supplementary Materials

The following supporting information can be downloaded at: https://www.mdpi.com/article/10.3390/ijms232315228/s1. References [94,95,96,97,98,99,100,101,102,103,104,105,106,107,108,109,110,111,112,113] are cited in the supplementary materials.
