# Supplementary material for: Endocannabinoid System Regulation in Female Rats with Recurrent Episodes of Binge Eating
Source: Int J Mol Sci. 2022 Dec 3;23(23):15228. doi: 10.3390/ijms232315228 (PMC9738776; doi:10.3390/ijms232315228)
Supplement: Supplementary file 1 [file ijms-23-15228-s001.zip › ijms-2057709-supplementary.pdf]

**Supplementary Information for "Endocannabinoid system regulation in female rats with recurrent episodes of binge eating"**

***Material and Methods***

*Binge Eating experimental procedure*

Twenty-four female Sprague Dawley rats were exposed (R) (or not exposed, NR) for 24 days to three 8-days cycles of food restriction (66% of their chow intake on days 1–4 and free feeding on days 5–8 of each cycle), during which they were given access to palatable food for 2 hours during the light cycle between 10:00 A.M. and 12:00 P.M. on days 5–6 and 13–14 of the first two cycles. Restriction to 66% of normal chow intake was defined for the first cycle based on the intake measured in the 6 days before the beginning of the experiment. For the second and third cycles, 66% restriction was defined based on the intake of restricted rats during the last 2 days of each 8-days cycle, when rats received only standard chow ad libitum. Daily standard food intake is shown in Figure S1.

On the first feeding test day (Day 25), half of the rats in each group were subjected to a 15-min frustration stress (S), consisting of the exposure to palatable food placed out of reach. The second half of rats in each group were not exposed to the stress manipulation (NS).

After 15 min of stress exposure, the palatable food was placed inside the cage for all rats and food intake was determined for 2 hours. Food intake was expressed as mean kilocalories ingested, normalized to rat body weights (kg) ± SEM.

To note, once assigned to one of these groups, the rats remained in that group throughout the experiments.

After 1 day off (free feeding day), rats were exposed or not to further 4 days of food restriction, followed by 4 days of free chow availability (see Figure 1a in the main text), in order to recover the previously lost body weight, before the access to palatable food for the second feeding test (Day 35). Following the identical schedule, the third (Day 45), and fourth (Day 55) feeding tests were performed, and in the fifth last test on Day 65, the rats were sacrificed immediately after stress, before eating palatable food, and the brains were collected. In accordance with our previous studies, binge eating (BE) behavior occurred only in Restricted and Stressed rats (R + S rats) with respect to the other groups. Since we [1,2] and others [3–7] previously observed that BE episode does not occur during the estrous phase, all female rats in each experimental group in this phase during the 4 feeding tests (day 25, 35, 45 and 55) were excluded from the statistical analysis. The hormonal fluctuations during the estrous cycle in non-testing days do not influence the development of the animal model [1,2]. At the beginning of the animal model, it is not possible to predict the precise estrus phase that the individual rat will have on the day of the feeding test for several reasons: some rats presented longer regular or irregular cycles [8–10], the synchronization of estrous cycle in all rats is highly unlikely [11–15] and food restrictions may influence the cyclicity of estrous cycle [16–20]. Considering the latter, Sprague–Dawley rats were chosen for our BE protocol, because this strain showed reliable cycle patterns during food deprivation [20].

The significantly increased palatable food intake ($P < 0.01$) in R + S rats compared to the other groups, in each feeding test, was maintained over time as shown in the Figure S2, since no significant interaction among the three conditions (food restriction" and "stress" and "time") was detected [$F(3, 47) = 0.57$ , $P > 0.05$].

*Diets*

Animals were offered standard rat food pellets, 4RF18, Mucedola, Settimo Milanese, Italy (2.6 kcal/g). The palatable food was a paste in texture, prepared by mixing Nutella (Ferrero, Alba, Torino, Italy) chocolate cream (5.33 kcal/g; 56%, 31% and 7% from carbohydrate, fat, and protein, respectively), grounded food pellets (4RF18, Mucedola, Settimo Milanese, Italy) and water in the following weight/weight percent ratio: 52% Nutella, 33% food pellets, 15% water. The palatable food diet had an energy density of 3.63 kcal/g.

*Chemical and reagents*

Analytical standards as N-arachidonoylethanolamide (AEA), N-arachidonoylethanolamide-d8 (AEA-d8), 2-arachidonoylglicerol (2-AG), 2-arachidonoylglicerol-d8 (2-AG-d8), Palmitoylethanolamide (PEA), Palmitoylethanolamide-d8 (PEA-d4), Oleoylethanolamide (OEA) and Oleoylethanolamide-d4 (OEA-d4) were

purchased from VinciBiochem s.r.l. (Vinci, FI, Italy). Water, methanol, acetonitrile, formic acid and chloroform, all UHPLC grade solvents, were purchased from VWR (Radnor, Pennsylvania, USA).

*Figures*

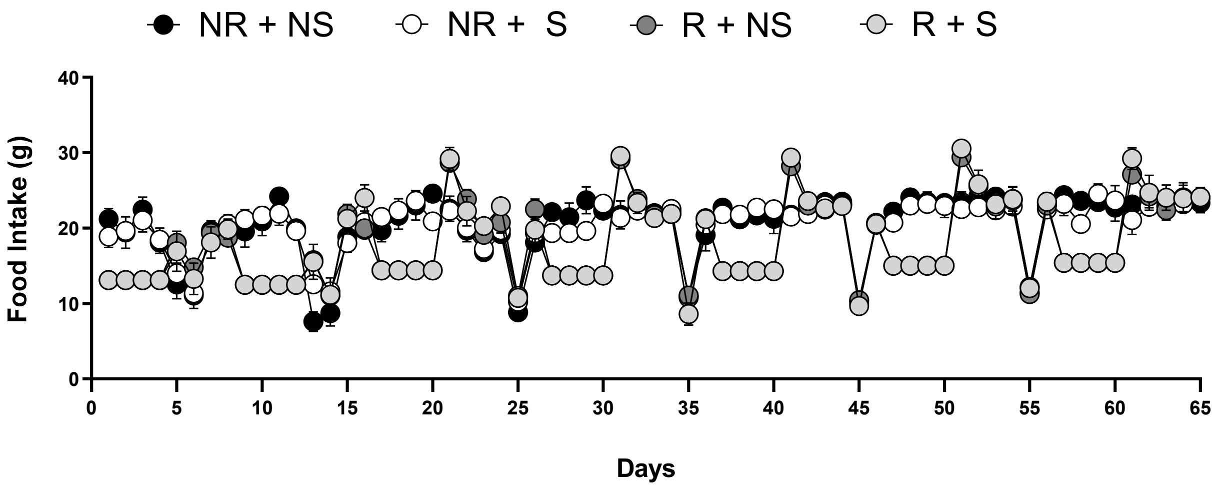


**Figure S1.** Schematic representation of standard food intake (grams) monitored throughout the entire duration of the study (from Day 1 to 65); n = 6 per group. Data are reported as Mean ± SEM.

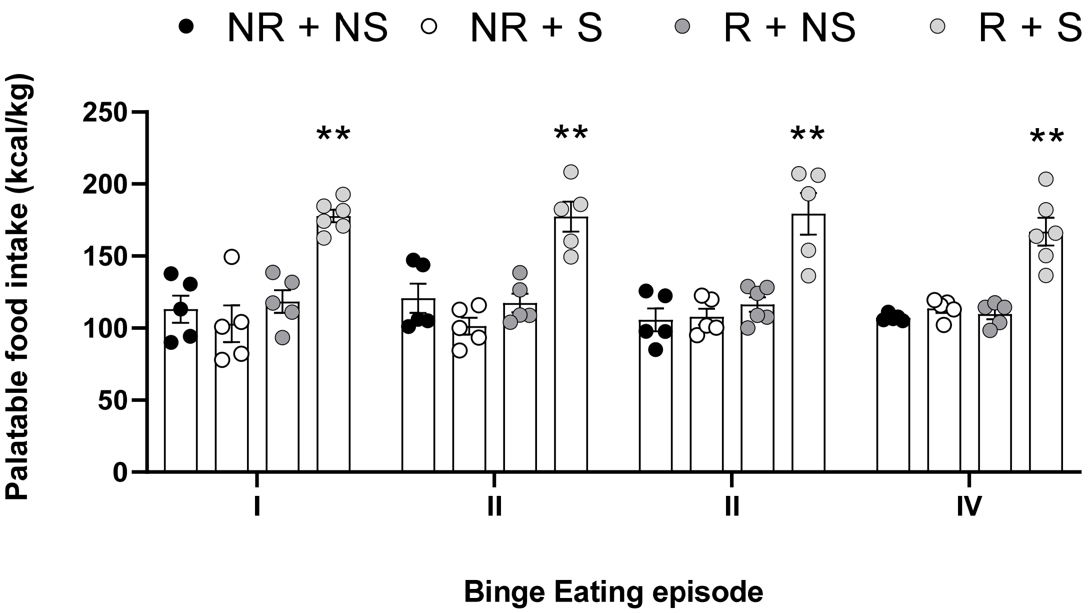


**Figure S2**. Mean ± SEM of cumulative palatable food intake (kcal/kg) during the four feeding tests in each experimental groups: NR + NS: Non restricted and Non stressed rats; NR + S: Non restricted and Stressed rats; R + NS: Restricted and Non stressed rats; R + S: Restricted and Stressed rats. * *P < 0.01 different from the other three groups in each feeding test; n = 5-6 per group. Data were analyzed by the mixed-effects model ANOVA, which included the between-subjects factors of "food restriction" and "stress" and the within-subjects factor "time" [food restriction $F(1, 20) = 65.95$ , $P < 0.0001$; stress $F(1, 20) = 38.64$, $P < 0.0001$; interaction food restriction x stress [$F(1, 20) = 52{,}25$, $P < 0.0001$; interaction food restriction x stress x time $F(3, 47) = 0.57$ , $P > 0.05$].

Bonferroni's post hoc tests were used to follow up on significant interaction or main effects (P < 0.05) from the factorial ANOVAs for each feeding test.

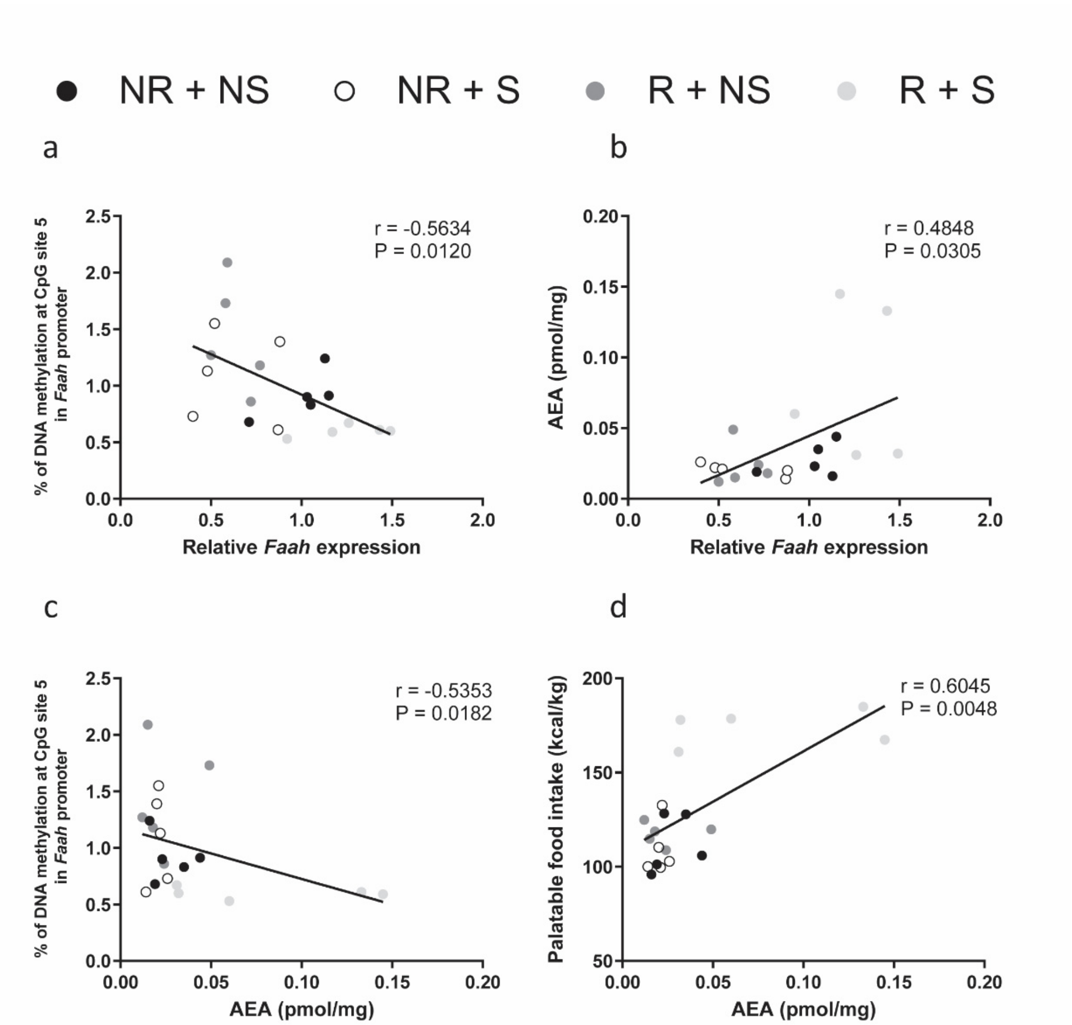


**Figure S3:** Correlation between *Faah* expression and DNA methylation level at CpG 5 (a) and AEA levels (B) and correlation between AEA levels and DNA methylation level at CpG 5 (c) and palatable food intake (d) ) in rats Hyp. Data were compared by Spearman's rank correlation coefficient, P and r values are reported.

*Tables*

**Table S1.** List of primers used for quantitative real-time RT–PCR.

| GENE | Forward | Reverse |
|---|---|---|
| ***β-Act*** | agatcaagatcattgctcctcct | acgcagctcagtaacagtcc |
| ***Gapdh*** | agacagccgcatcttcttgt | cttgccgtgggtagagtcat |
| ***Cnr1*** | ttccaccgtaaagacagccc | tccacatcaggcaaaaggcc |

| | | |
|---|---|---|
| ***CB2*** | ttgaccgatacctatgtctgtgc | tgctttccagaggacataccc |
| ***Faah*** | atggaagtcctccaagagc | tagagctttcaggcatagcg |
| ***Nape-pld*** | tgtcccgggttccaaagaggagc | accatcagcgtcgcgtgtcc |
| ***Dagl*** | attctctccttcctcctgc | atttgggcttggtgcttcg |
| ***Magl*** | atgttgaagaggctggacatgc | atgcagattccggattggc |

**Table S2.** Details of sequences and primers employed during the analysis of DNA methylation

| GENE | PRIMERS (5′ – 3′) | SEQUENZE TO ANALYZE* | GENOMIC LOCATION (Rat mRatBN7.2) |
|---|---|---|---|
| *Cnr1* | F: agaagggtaagatttggtatagtg<br>R-biotin: aactatacaactaaataaacaccacatta<br>S: gtggagtttgggaatagttt | …tagcttttc**cg**cctccc**cg**ccccct**cg**atactggccag tggtccccaggtgtcctac**gcg**ga gtt**cg**tgttc**cg**tgttccagaaga… | Chromosome 5: 48410585 - 48410669 |
| *Faah* | Included in the assay PM00559699 | …cc**gg**cc**cg**cccc**cg**gtgtttttgag ggtgccca**cgcg**ga… | Chromosome 5: 129498868 - 129498907 |
| *Dagl* | Included in the assay PM00453670 | **…cg**ggcag**cg**tgatttttc**cg**gg**cg**a… | Chromosome 1: 206946937 - 206946964 |
| *Magl* | Included in the assay PM00551978 | …gg**cg**gggaggg**cgcg**gaccc**g**gtggt gctgccctg**cg**gc**cgcg**a… | Chromosome 4: 121192795 - 121192838 |

*Bold text = CpG sites analysed; Lowercase letters = 5' upstream sequence.

**Table S3.** Primer sequences used for histone modification experiments.

| **Gene** | **Forward** | **Reverse** |
|---|---|---|
| ***Cnr1*** | gagcaaatcgtgctaatgtgg | agctaggggcacattcagtaaa |
| ***Faah*** | gttgaagctgtggggtgtct | cgtgccagcgttttaagg |
| ***Dagl*** | gggcaaagtggatacagcac | tacgttgcatcgccctcc |
| ***Magl*** | tctggaaaagtggcgacatga | agtatcagcgccaggcaaac |

*References*


1. Alboni, S.; Micioni Di Bonaventura, M.V.; Benatti, C.; Giusepponi, M.E.; Brunello, N.; Cifani, C. Hypothalamic Expression of Inflammatory Mediators in an Animal Model of Binge Eating. *Behav. Brain Res.* **2017**, *320*, 420–430, doi:10.1016/j.bbr.2016.10.044.
2. Micioni Di Bonaventura, M.V.; Lutz, T.A.; Romano, A.; Pucci, M.; Geary, N.; Asarian, L.; Cifani, C. Estrogenic Suppression of Binge-like Eating Elicited by Cyclic Food Restriction and Frustrative-

Nonreward Stress in Female Rats. *Int. J. Eat. Disord.* **2017**, *50*, doi:10.1002/eat.22687.
3. Culbert, K.M.; Racine, S.E.; Klump, K.L. Hormonal Factors and Disturbances in Eating Disorders. *Curr. Psychiatry Rep.* **2016**, *18*, 65, doi:10.1007/s11920-016-0701-6.
4. de Ávila, C.; Chometton, S.; Calvez, J.; Guèvremont, G.; Kania, A.; Torz, L.; Lenglos, C.; Blasiak, A.; Rosenkilde, M.M.; Holst, B.; et al. Estrous Cycle Modulation of Feeding and Relaxin-3/Rxfp3 MRNA Expression: Implications for Estradiol Action. *Neuroendocrinology* **2021**, *111*, 1201–1218, doi:10.1159/000513830.
5. Edler, C.; Lipson, S.F.; Keel, P.K. Ovarian Hormones and Binge Eating in Bulimia Nervosa. *Psychol. Med.* **2007**, *37*, 131–141, doi:10.1017/S0033291706008956.
6. Klump, K.L.; Culbert, K.M.; Sisk, C.L. Sex Differences in Binge Eating: Gonadal Hormone Effects Across Development. *Annu. Rev. Clin. Psychol.* **2017**, *13*, 183–207, doi:10.1146/annurev-clinpsy-032816-045309.
7. Lamontagne, S.J.; Wilkin, M.M.; Menard, J.L.; Olmstead, M.C. Mid-Adolescent Stress Differentially Affects Binge-like Intake of Sucrose across Estrous Cycles in Female Rats☆. *Physiol. Behav.* **2021**, *228*, 113194, doi:10.1016/j.physbeh.2020.113194.
8. Marcondes, F.K.; Bianchi, F.J.; Tanno, A.P. Determination of the Estrous Cycle Phases of Rats: Some Helpful Considerations. *Braz. J. Biol.* **2002**, *62*, 609–614, doi:10.1590/s1519-69842002000400008.
9. Freeman, M. Neuroendocrine Control of the Ovarian Cycle of the Rat. In *Knobil and Neill's Physiology of Reproduction*; Elsevier, 2006; pp. 2327–2388.
10. Nequin, L.G.; Alvarez, J.; Schwartz, N.B. Measurement of Serum Steroid and Gonadotropin Levels and Uterine and Ovarian Variables throughout 4 Day and 5 Day Estrous Cycles in the Rat. *Biol. Reprod.* **1979**, *20*, 659–670, doi:10.1095/biolreprod20.3.659.
11. Marashi, V.; Rülicke, T. The Bruce Effect in Norway Rats. *Biol. Reprod.* **2012**, *86*, 1–5, doi:10.1095/biolreprod.111.093104.
12. Sharp, P.; Villano, J.S. *The Laboratory Rat*; CRC Press, 2012; ISBN 9780429152405.
13. Lohmiller, J.J.; Swing, S.P.; Hanson, M.M. Reproduction and Breeding. In *The Laboratory Rat*; Elsevier, 2020; pp. 157–179.
14. Shirley, B. Partial Synchrony of the Oestrous Cycles of Rats Introduced to a New Environment. *J. Endocrinol.* **1978**, *77*, 195–202, doi:10.1677/joe.0.0770195.
15. Schank, J.C. Do Norway Rats (Rattus Norvegicus) Synchronize Their Estrous Cycles? *Physiol. Behav.* **2001**, *72*, 129–139, doi:10.1016/s0031-9384(00)00395-4.
16. Howland, B.E. Effect of Restricted Feed Intake on LH Levels in Female Rats. *J. Anim. Sci.* **1972**, *34*, 445–447, doi:10.2527/jas1972.343445x.
17. Knuth, U.A.; Friesen, H.G. Starvation Induced Anoestrus: Effect of Chronic Food Restriction on Body Weight, Its Influence on Oestrous Cycle and Gonadotrophin Secretion in Rats. *Acta Endocrinol. (Copenh).* **1983**, *104*, 402–409, doi:10.1530/acta.0.1040402.
18. Lintern-Moore, S.; Everitt, A. V The Effect of Restricted Food Intake on the Size and Composition of the Ovarian Follicle Population in the Wistar Rat. *Biol. Reprod.* **1978**, *19*, 688–691, doi:10.1095/biolreprod19.3.688.
19. Seki, M.; Yamaguchi, K.; Marumo, H.; Imai, K. Effects of Food Restriction on Reproductive and Toxicological Parameters in Rats--in Search of Suitable Feeding Regimen in Long-Term Tests. *J. Toxicol. Sci.* **1997**, *22*, 427–437, doi:10.2131/jts.22.5_427.
20. Tropp, J.; Markus, E.J. Effects of Mild Food Deprivation on the Estrous Cycle of Rats. *Physiol. Behav.* **2001**, *73*, 553–559, doi:10.1016/s0031-9384(01)00487-5.
